# Zebrafish Modeling of Autism Spectrum Disorders, Current Status and Future Prospective

**DOI:** 10.3389/fpsyt.2022.911770

**Published:** 2022-07-14

**Authors:** Akram Tayanloo-Beik, Shayesteh Kokabi Hamidpour, Mina Abedi, Hamide Shojaei, Mostafa Rezaei Tavirani, Nazli Namazi, Bagher Larijani, Babak Arjmand

**Affiliations:** ^1^Cell Therapy and Regenerative Medicine Research Center, Endocrinology and Metabolism Molecular-Cellular Sciences Institute, Tehran University of Medical Sciences, Tehran, Iran; ^2^Proteomics Research Center, Shahid Beheshti University of Medical Sciences, Tehran, Iran; ^3^Diabetes Research Center, Endocrinology and Metabolism Clinical Sciences Institute, Tehran University of Medical Sciences, Tehran, Iran; ^4^Endocrinology and Metabolism Research Center, Endocrinology and Metabolism Clinical Sciences Institute, Tehran University of Medical Sciences, Tehran, Iran

**Keywords:** autism spectrum disorders, animal modeling, *Danio rerio*, drug development, drug discovery, zebrafish

## Abstract

Autism spectrum disorder (ASD) refers to a complicated range of childhood neurodevelopmental disorders which can occur *via* genetic or non-genetic factors. Clinically, ASD is associated with problems in relationships, social interactions, and behaviors that pose many challenges for children with ASD and their families. Due to the complexity, heterogeneity, and association of symptoms with some neuropsychiatric disorders such as ADHD, anxiety, and sleep disorders, clinical trials have not yielded reliable results and there still remain challenges in drug discovery and development pipeline for ASD patients. One of the main steps in promoting lead compounds to the suitable drug for commercialization is preclinical animal testing, in which the efficacy and toxicity of candidate drugs are examined *in vivo*. In recent years, zebrafish have been able to attract the attention of many researchers in the field of neurological disorders such as ASD due to their outstanding features. The presence of orthologous genes for ASD modeling, the anatomical similarities of parts of the brain, and similar neurotransmitter systems between zebrafish and humans are some of the main reasons why scientists draw attention to zebrafish as a prominent animal model in preclinical studies to discover highly effective treatment approaches for the ASD through genetic and non-genetic modeling methods.

## Introduction

Autism spectrum disorder (ASD) is defined as a complex and lifelong neurodevelopmental disorder encompassing a range of conditions that cause challenges with social interactions and interpersonal communications as well as atypical patterns of behaviors, activities, and interests (1, 2), which manifests in childhood (3). The term “autism” was first coined by a psychiatrist known Eugen Bleuler in the early twentieth century (4). Many years later, Leo Kanner introduced the term “infantile autism” as a more accurate definition of autism to describe children with an inherent inability to social and verbal communication with others. One of the features that Kanner emphasized was the obsessive insistence of autistic children on maintaining the same. In years afterward, as studies progressed, another group of similar diseases was discovered by scientists that did not meet all the criteria presented by Kanner. Since then, there have been changes in the definition of autism disease (5). In 2013, the updated version of the book “Diagnostic and Statistical Manual of Mental Disorders (DSM-5),” has defined the diagnostic criteria of ASD as follows:

1 Challenges with social interactions and interpersonal communications:
  1.1 Difficulties with socio-emotional interactions.
  1.2 Difficulties with non-verbal communication skills.
  1.3 Weaknesses in establishing, maintaining, and expanding relationships.
2 Atypical patterns of behaviors, activities, and interests:
  2.1 Repetitive, restricted, and stimming behavioral patterns.
  2.2 Insisting on performing regular routines and displaying ceremonial patterns, whether they are verbal or non-verbal behaviors.
  2.3 Interests accompanied by unusual focus or intensity.
  2.4 Displaying sensory over-responsivity or sensory under-responsivity and atypical sensory interests (1).

Studies imply that in the 1970s autism prevalence rate was very low worldwide (about 0.05% of children). However, in recent decades, the autism prevalence rate has risen dramatically (about 0.9–1.5% of children), which is sometimes referred to as the “autism epidemic” (6). Accordingly, a further increase in the prevalence rate of ASD can impose a heavy burden on more individuals and families (in terms of high costs of care, more stress and anxiety, reduced social support, etc.) (7) as well as government entities such as the healthcare and economic systems (8). In recent years, many efforts, such as pharmacological approaches (e.g., risperidone and aripiprazole) and non-pharmacological approaches (e.g., behavioral, developmental, and educational methods), have been made to treat patients. However, those approaches can only alleviate the symptoms. Hence, it can be concluded that no definite treatment has been found for ASD that results in a notable outcome (9). Despite the mentioned approaches toward improving the patient’s quality of life, the need to discover many effective treatment methods remains noteworthy. Hence, achieving a deep knowledge of the mechanisms underlying ASD can be a big step forward in finding new therapeutic approaches for the patients. In this regard, preclinical studies have a great contribution to bridging the theoretical knowledge to the clinical results in drug discovery and development platforms, in which animal models are an indispensable part. In recent decades, a wide range of animals has been studied in this field, among which mice are notable (10). Although mouse models are used traditionally as a prominent model in neurological disorders due to features such as the high similarity between nervous system function and related genes to those of humans as well as the possibility of genetic manipulation to model the diseases, they have their limitations and shortcomings (11–14). For instance, laboratory mice are mainly active during night-time. In addition, they are susceptible to changes in environmental conditions (e.g., temperature, light, and sound). It is also worth mentioning that mouse model-based experiments require considerable cost and effort (15). Moreover, monitoring and examining the social behaviors of mice takes up a great deal of time, whether in real-time laboratory experiments or video recorded, which can pose difficulties to translational research from bench to bedside (16). Non-human primate (NHP) has been also used as another animal model to simulate the behavioral part of ASD phenotype due to the similarity between their neurological circuits associated in behavior to those of humans. Moreover, NHP can open new doors to the study of immunological factors involved in the development of ASD (17). However, developing ASD NHPs models, which fall into the category of complex organisms in terms of structure and function, requires strict ethical considerations (18). In addition to the above, some animal models, such as Prairie vole, Songbird, Drosophila, Aplysia, and *C. elegans*, have been studied as candidate models for the development of ASD. However, even these models cannot properly demonstrate the complexities of ASD (17). Therefore, achieving more comprehensive information on the pathophysiology of ASD highlights the need to seek efficient animal models to pave the way for discovering more effective therapeutic approaches. In recent years, there has been a great interest in using zebrafish as a promising animal model in neurological disorders studies due to their outstanding features. Regarding the neurological disorders, the salient features of zebrafish can be examined in two categories:

1. General features: Zebrafish embryos develop independently of the maternal fish. In addition to the fast embryonic development, the zebrafish embryos have transparent bodies, which can facilitate directly monitoring of many biological and neurobiological processes. Moreover, the short life cycle and many offspring per reproductive period represent zebrafish as a convenient model for high throughput screening (HTS) (19). Furthermore, the small size of body, the low maintenance costs, and easy housing of a large number of them in a small aquarium are other advantages of zebrafish, which have made them as a model of interest in drug discovery and development pipelines (20).
2. Specialized features related to the nervous system: zebrafish show high a resemblance to mammals in terms of brain morphology and nervous system as well as neural and signaling pathways (19, 21). Additionally, zebrafish display excellent potential for the development of specific social attitudes that play an important role in simulating ASD phenotypes (22). Regarding genetic studies, it is worth mentioning that there is high orthology between zebrafish and human genes, which leads to the transcription of proteins with similar goals and ultimately similar functions of different body systems, especially the nervous system (21). Hereupon, this review will initially discuss the pathophysiology of ASD and reveal the related pharmacological and non-pharmacological therapeutic approaches. Then, this review is focused on zebrafish as a prominent animal model in ASD modeling. Finally, the studies on genetic and non-genetic modeling methods are described.

## An Overview on Pathophysiology of Autism Spectrum Disorder

**FIGURE 1:**
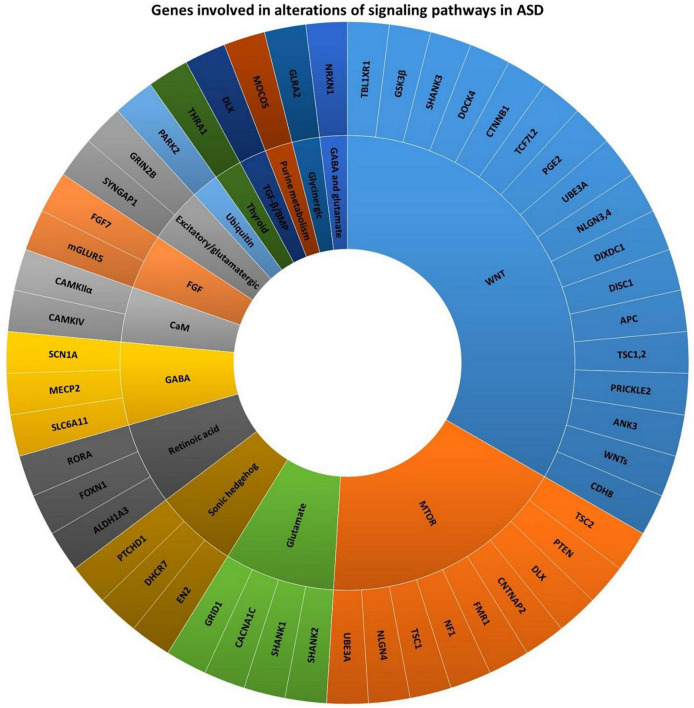
Genes involved in alterations of signaling pathways in ASD (26, 34).

Autism spectrum disorder refers to a group of early childhood neurodevelopmental disabilities that lasts a lifetime and results in impaired social behavior, difficulties in communication, and repetitive type compulsive behaviors (23). Studies imply that ASD is a multifactorial disease (24, 25). Remarkable advances in the knowledge of ASD risk factors have revealed that genetic mutations are among the main factors involved in alterations of the cell and molecular processes (Figure 1) (26). According to preclinical and clinical studies, over 800 ASD-related genes have been reported (27), of which about 100 genes are significantly correlated with the development of autism-related behaviors (28). For instance, AVPR1a, DISC1, DYX1C1, ITGB3, SHANK3, SLC6A4, RELN, and RPL10 are among the genes which are implicated in the metabolic alterations related to the brain in ASD patients (29). To promote the research in this field, genetic studies related to ASD have been conducted in three main areas:

1. Monozygotic (MZs) and dizygotic (DZs) twins: In genetic studies, examining the inheritance of the disease in twins can be considered one of the important and practical approaches. In ASD condition, comparisons of the concordance rate between MZs twins and DZs twins indicate that if one of the MZs twins has autism, the risk of developing the disease in the other twin is high. However, due to the low rate of gene sharing in DZs twins, the risk of developing the disease in the other twin is lower than in MZs condition. Nevertheless, a high impact of the common prenatal environment on the development of autism has been reported compared to genetic factors in twins.
2. First-degree relatives: In this case, a term called “The broad autism phenotype (BAP)” states that cognitive and behavioral changes associated with autism are manifested more closely in individuals without autism, who are categorized as first-degree relatives of autistic people. In other words, the incidence of autism in a family member can be associated with increases in the odds of ASD-related mild impairments in social and communication skills development in non-autistic relatives (30).
3. Rare genetic syndromes comorbidity associated with autism: Individuals with rare genetic syndromes may be more likely to present degrees of ASD-like characteristics. For instance, Fragile X syndrome, Timothy syndrome, Rett syndrome, Tuberous sclerosis, Hamartoma tumor syndrome, Prader-Willi syndrome, Angelman syndromes, DiGeorge syndrome, and neurofibromatosis type 1 are among the most common known genetic syndromes associated with ASD (31). It should also be noted that, as the incidence of co-occurrence between the expressed genetic syndromes and ASD are different, the demonstration of characteristics associated with ASD in these patients can also be varied (30).

On the other hand, non-genetic factors such as environmental factors (e.g., exposure to infections, air pollutants, pesticides, and psychological stress, anti-epileptic drug administration, and folate deficiency) (32) or epigenetic factors (e.g., changes in DNA methylation patterns and microRNAs expression) also play an important role in the development of ASD symptoms (27).

To facilitate studies in this field, the ASD risk factors can be also classified into three other general categories, including (1) prenatal factors (e.g., advanced maternal age and race/ethnicity), (2) perinatal factors (e.g., maternal hypertension, overweight, and diabetes, infections during pregnancy, cesarean section delivery, and preterm birth), and (3) postnatal factors (e.g., postpartum hemorrhage, and abnormal brain growth). It should also be noted that exposure to air pollution in any of the three stages can increase the risk of ASD development (24).

After achieving a comprehensive understanding of known risk factors involved in autism, an important question arises. How do these risk factors give rise to autism? In response to this question, studies suggest that the effect of risk factors in autism is greater on early brain development. In other words, anatomical study of people with ASD reveals changes in the structure of their brains compared to healthy people (33). Hence, to promote the study in this field, a better understanding of differences in brain development in ASD patients’ needs to be highlighted, that will discuss in more detail in the following sections.

### Autistic Brain Characteristics

Research has shown that ASD individuals have some abnormalities with brain connectivity often involving frontal or occipital regions. It has been shown that the connectivity between different brain regions (“long-range” connectivity) has been decreased in ASD patients, whereas the connectivity within some specialized regions (“short-range” connections) has been increased. “long-range” connectivity is important for global processing of the incoming information and integrating them. However, “short-range” connections are associated with details of the incoming information. Also, it has been demonstrated that in ASD patients, the left hemisphere plays a greater role in processing the information compared to the right hemisphere. Notably, the left hemisphere is more related to details, whereas the right hemisphere is more related to global and integrated processing. Putting abnormal connectivity and left lateralization, together can explain impairment in facial recognition and spatial skills in ASD individuals (35). Brain volume in ASD patients is another issue that has been addressed in investigations. The brain volume of children with ASD develops rapidly, particularly in the frontal and temporal lobes. But this growth stops at older ages. In addition, the brain volume of adults with ASD is unchanged or reduced compared to the control group (36). To achieve a better understanding of the mechanisms underlying ASD, it needs to zoom in and broaden our perspective to the level of the constituent parts of the brain. In this regard, physiological studies imply that the disturbance in four social areas of the brain, including the amygdala, the orbitofrontal cortex (OFC), the temporomandibular cortex (TPC), and the insula, have key roles in triggering the ASD symptoms (37). In addition to above, dysfunctionality and defect in some regions such as frontotemporal lobe, front parietal cortex, basal ganglia, anterior cingulate cortex (ACC), and hippocampus can have a great contribution to not only the development of ASD but also some disorders such as anxiety and schizophrenia (36). In addition, changes in the volume of white matter in different areas of the brain in people with ASD have been observed compared to normal populations, which may indicate a defect in oligodendrocytes and myelination in some areas of the central nervous system (CNS) under the influence of some genes and growth factors such as Olig1, Olig2, PGFRA, Sox10, Gpr17, EGF, IGF-1, and Nkx2-2. This evidence also suggests that the pattern of abnormal myelination in the brains of people with ASD varies from year to year. For example, in the first 15 years of life, a decrease in the amount of the myelin covering the axons of the arcuate, occipitofrontal fasciculus, and external capsule can be observed. While the amount of myelin in some areas, such as the hippocampus and fusiform gyrus, is highly more than normal. Some of these areas tend to normalize over time, but others, such as the cerebellum and the internal capsule remain hypo-myelinated (38). The pathophysiology of ASD can also be traced to the level of the cells that comprise nervous tissue. For example, microglia cells are dedicated as resident macrophages in the CNS that functions as an innate immune cells to respond to pathogens and neurological injuries. In case of inflammation, nerve damage, and tissue hemostasis disturbance, microglia cells transit from the rest state to the active state, which is mainly accompanied by changes in the genetic and cellular process as well as morphological alterations (39). According to ASD clinical investigations, the activity of microglia in three parts of the brain, including white matter, cortical regions, and cerebellum of postmortem are significantly increased. In addition, an increase in microglia density can be observed in the dorsolateral prefrontal, the fronto-insular, and the visual cortex. Morphologically, the soma size, the filopodia extension, and the dynamic process extension and retraction are also increased in ASD patients’ fronto-insular and visual cortex. However, the observations imply that the granule and Purkinje cell numbers as well as numbers of axons located in the Purkinje cell layer (PCL) in the ASD cerebellum decreased. Also, researches on the brains of ASD patients show that the microglia and neurons in the dorsolateral prefrontal cortex (PFC) are located very close to each other (more than normal), which can justify the over-destruction of healthy neurons and the demolition of dysfunctional synapses by microglia. It should also be noted that, although the level of ionized calcium-binding adapter molecule 1 (IBA1) decreases in the cerebellum, the hyperactivation of three genes, including *TREM2*, *DAP12/KARAP*, and *CX3CR1* can be observed in PFC (40). Defects in synaptic pruning and disruption of the brain’s excitatory vs. inhibitory (E/I) ratio (effective factor in the development of social withdrawal, language communication difficulties, and restrictive interests or repetitive behaviors in ASD) are some of the microglia dysfunctionalities which can be developed in the brain of ASD patients (41). In addition to microglia, astrocytes have been studied as another important group of cells in the ASD pathogenesis mechanism. This heterogeneous group of cells, which are resident in CNS, have a crucial role in different biological process like maintenance of extracellular ion balance, the formation of new neurons and synapses, and regulation of blood-brain barrier integrity. In case of CNS lesions, astrocytes can trigger the inflammatory state of the CNS as a result of increased expression of glial fibrillary acidic protein (GFAP) (42). Additionally, compared to microglia, astrocytes express more types and numbers of excitatory amino acid transporters (EAATs), which are responsible for the glutamate uptake and release. Therefore, it can be concluded that astrocytes play a more important role than microglia in glutamate homeostasis in the CNS (40). In addition to the stated roles for astrocytes, attention to the interaction between astrocytes and microglia is necessary. In a simple expression, astrocytes activate microglia by secreting ATP. Conversely, microglia affect the activity of astrocytes by secreting cytokines and chemokines. The mentioned cross-talk between astrocytes and microglia is involved in regulating synapse function, maintaining synaptic plasticity, and mediated immune response in the body. Therefore, in ASD condition, any abnormality in these type of cells can lead to the emergence of ASD symptoms such as unusual behaviors and cognitive problems in patients. Moreover, the level of GFAP in the PFC and cerebellum increases dramatically in case of ASD (40).

### Relationship Between Gastrointestinal Tract and Brain in Autism Spectrum Disorder

In recent years, specific alterations in gastrointestinal functions have been explored as a new perspective on the pathophysiology of patients with autism. Mainly, ASD patients suffer more digestive problems in comparison with non-ASD people, one of the most common of which is constipation. It has also been shown that digestive problems can lead to displaying some autistic behaviors such as aggression, anger, and lack of proper sleep in patients. In contrast, sometimes diarrhea and bloating are among the causes of atypical behaviors in ASD patients. The cause of diarrhea and bloating problems can be found in the metabolism and absorption impairments of carbohydrates in the patient’s small intestine. More specifically, impaired absorption of mono- and disaccharides in the small intestine causes the transfer of these carbohydrates to the large intestine. As a result, competition between polysaccharide-degrading bacteria and mono- and disaccharide-degrading bacteria increases, which in turn leads to the production of specific bacterial compounds as well as diarrhea and bloating. Microbial dysbiosis is another disorder observed in ASD patients. In addition, cellular and molecular studies indicate that mitochondrial dysfunctionality and high susceptibility of enterocytes to oxidative stress, which can eventually lead to digestive problems in ASD patients. ASD condition is also accompanied by increased intestinal permeability. It is probably due to two reasons: intestinal barrier-forming proteins are less expressed, and conversely, pore-forming proteins of tight junctions are overexpressed in the cells. As a result, these alterations can facilitate the transition of bacterial metabolites through the intestinal barrier, which can trigger inflammation of the brain. In addition, ASD patients show the presence of specific bacterial metabolites [e.g., short-chain fatty acids (SCFAs), indoles, and lipopolysaccharides (LPS)] in the analysis. The production of such bacterial products can trigger some symptoms in ASD patients due to the connection between the intestine and the nervous system. One of the main evidences for the connection between the gastrointestinal and the nervous system is the presence of vagal fibers. But the main question is how this bridge of communication works. In response to this question, evidence shows that the afferent vagal fibers have a synapse with a type of cells resident in intestinal epithelium called enteroendocrine cells, which have a crucial role as epithelial biosensors and can be stimulated by lumen nutrients and bacteria. Therefore, any production of specific and unusual metabolites can lead to an effect on the gut-microbiota-brain axis. Also, high levels of neurotransmitters such as serotonin, inhibitory neurotransmitter gamma-aminobutyric acid (GABA), and noradrenaline, which are also produced by certain species of bacteria, can affect the CNS through intestinal cells and development of various symptoms of ASD in patients (43).

### Neurotransmitter Systems in Autism

Neurotransmitters are a wide range of chemical substances, which can boost, balance, and transfer neurological signals from a neuron to the target cell to maintain the proper nervous system and the whole body functions at the end. In addition to transmitting nerve impulses, neurotransmitters play an important role in vital processes such as the growth and differentiation of neurons as well as the development of neural circuits. Due to the vital role of neurotransmitters in the nervous system, it can be understood that any defect or problem in this system can lead to emerging various diseases, such as neurodevelopmental disorders (44). Over the past years, based on the efforts to scrutinize the mechanisms underlying ASD, it has been realized that the neurotransmitter system dysfunctionality is one of the causes of symptoms development in autistic patients. Among the wide range of neurotransmitters, the altered GABAergic system has been considered one of the main reasons linked to ASD pathophysiology, which can lead to cognitive impairments. Conventionally, GABA is involved in vital processes such as the differentiation and maturation of neurons, cell migration, and neuronal-network wiring with an inhibitory influence on the human cortex. However, in ASD condition, the expression of GAD65 and GAD67 decrease, which ultimately lead to GABAergic dysfunctionality *via* disrupting the conversion of glutamate to GABA. Additionally, in ASD patients, GABA platelet, GABAA, and GABAB levels have been reported lower than normal, and some of these defects are probably related to genetic mutations. In addition to GABA, glutamate (the anion of glutamic acid) as an excitatory neurotransmitter is implicated in ASD pathophysiology. This type of neurotransmitter plays an important role in vital processes such as neuronal development, memory, cell migration, and synaptic plasticity under normal physiological conditions. However, in ASD patients, the glutamatergic system encounters dysfunctionalities. However, it is not clear exactly whether the dysfunctionality is due to hypoglutamatergic or hyperglutamatergic states. To scrutinize these hypotheses, various preclinical and clinical assessments have been conducted. Nevertheless, due to the complexity of the ASD pathophysiology, some studies place more emphasis on the precedence of the hypoglutamatergic hypothesis than on the hyperglutamatergic state, and some studies vice versa. On the whole, it should be emphasized that whether the disturbance of the glutamatergic system is caused by a hypoglutamatergic or hyperglutamatergic state, it will eventually result in the development of ASD symptoms. Similar to glutamate, there are two possible hypotheses for the role of serotonin in ASD, which include hyposerotonin and hyperserotonin states. Although there are some controversies on the validity of one of the hypotheses as the main factor involved in ASD development, observations suggest that some ASD patients get involved with low serotonin levels in the brain as well as high serotonin levels in the blood. In addition to the above, studies at the level of neurotransmitters show abnormalities in the dopaminergic, and subsequently the catecholaminergic systems. Additionally, tests performed on patients’ blood, urine, and cerebrospinal fluid (CSF) showed that patients with ASD had high serum norepinephrine levels (45). In addition to the neurotransmitter systems mentioned above, preclinical and clinical studies have shown evidence of an important role of cholinergic system disturbance in the appearance of some symptoms such as repetitive behaviors, difficulties with social communication skills, cognitive impairment, and unusual attention in ASD patients. Observations on the brain of ASD patients indicate that the cholinergic basal forebrain neurons differs in number and structure manner. In addition, evidence show the unusual nicotinic-cholinergic receptor (nAChR) subunits in some brain regions such as cerebellum, neocortex, striatum, and thalamus, some of which have been the result of mutations in genes *CHRNA7* and *CHRNB2*. It is worth mentioning that the regulation of some of the neurotransmitters functions are under control of histaminergic system. Similar to the other neurotransmitter systems, any disturbance in histaminergic system can also develop ASD symptoms such as cognitive problems. Therefore, understanding the mechanisms underlying histaminergic system can pave the way for discovery new therapeutic approaches to improve ASD symptoms (46).

### Diagnosis and Treatment Approaches for Autism Spectrum Disorder Patients

Accurate diagnosis of ASD is at the top challenges list of the patient’s treatment. Although evidence suggests that some methods such as DNA analysis for fragile X syndrome, regular karyotyping, and auditory testing can be complementary approaches to ASD diagnosis, there are no accurate blood tests or imaging techniques to determine the disease (47). In addition, the complexity of the disease and the presence of some identical symptoms between ASD and other psychological disorders can pose problems in early and accurate diagnosis. Therefore, most diagnostic approaches are based on caregiver’s and patients’ reviews, the family history assessment, and real-time patient behaviors investigations. In this regard, some approaches such as Childhood Autism Rating Scale (CARS), The Developmental, Dimensional, and Diagnostic Interview (3di), The Autism Spectrum Disorder-Observation for Children (ASD-OC), The Autism Diagnostic Interview-Revised (ADI-R), The Asperger Syndrome Diagnostic Interview (ASDI), The Diagnostic Interview for Social and Communication Disorders (DISCO), and The Autism Spectrum Disorder–Diagnosis Scale for Intellectually Disabled Adults (ASD-DA) are considered as common diagnostic approaches, which can be applied for ASD patients. After the accurate diagnosis of the disease, the most important issue is to apply effective treatment approaches. As mentioned in the previous sections, there is no definitive treatment for ASD patients. However, applying some pharmacological and non-pharmacological interventions can have a major contribution to relieving symptoms and subsequently improving the quality of life of patients with ASD, which have been discussed in more detail in Table 1 (48).

**TABLE 1:** Current therapeutic approaches for ASD patients.

Treatment types	Treatment subtypes	Examples of drugs	Advantages	Side effects	References
Pharmacological treatments	Psychostimulants	Methylphenidate	Impulsivity**↓** Hyperactivity↓ ADHD symptoms of autistic patients↓	Appetite ↓ Problem in the digestive tract*uparrow* Headache *uparrow*	(48, 49)
		Amphetamines	Impulsivity↓ Hyperactivity↓ ADHD symptoms of autistic patients↓	Irritability*uparrow* Sleep disorders, anxiety, intensification of behaviors in mixed amphetamine salts prescription	(48, 50)
	Atypical antipsychotic drugs	Risperidone	Hyperactivity↓ Repetitive behaviors↓ Aggression↓ Social withdrawal↓ Anxiety↓ Nervousness↓ Depression↓ Irritability↓	Weight gain*uparrow* Fatigue*uparrow* Drowsiness*uparrow* Dizziness↓	(48)
		Aripiprazole	Irritability↓	Sedation*uparrow* Drooling*uparrow* Tremor*uparrow*	(48)
		Quetiapine	Aggression↓ Sleep disturbance↓	Sedation*uparrow* Aggression*uparrow* Weight gain*uparrow*	(48, 51)
		Ziprasidone	Aggression↓ Agitation↓ Irritability↓	Weight loss↓	(48)
		Olanzapine	Anger↓ Irritability↓ Anxiety↓ Hyperactivity↓ Social withdrawal↓ Language usage*uparrow*	Weight gain*uparrow* Appetite changes Impaired insulin sensitivity*uparrow*	(48)
	Antidepressant drugs	Fluoxetine	Repetitive behaviors↓ Anxiety↓	Restlessness*uparrow* Agitation*uparrow* Hyperactivity*uparrow* Problems in appetite Sleep disturbance*uparrow*	(48, 52)
		Sertraline	Irritability↓ Anxiety↓ Aggressive behaviors↓ Repetitive behaviors↓		(48)
		Citalopram	Anxiety↓ Aggression↓ Repetitive behaviors↓ Irritability↓	Hyperactivity*uparrow* Insomnia*uparrow* Diarrhea*uparrow*	(48)
		Escitalopram	Impulsivity↓ Improving Psychosocial behaviors*uparrow*	Hyperactivity*uparrow* Aggression*uparrow* Irritability*uparrow*	(48)
		Fluvoxamine	Anxiety↓ Repetitive behaviors↓ Aggression↓	Akathisia*uparrow* Agitation*uparrow* Sleep difficulties*uparrow* Headaches*uparrow* Appetite changes	(48)
	Alpha-2 adrenergic receptor agonists	Clonidine	Hyperarousal behaviors↓ Social interactions*uparrow* Irritability↓ Hyperactivity↓ Sleep initiation latency↓ Insomnia↓ Aggressive behaviors↓	Sleepiness*uparrow* Sedation*uparrow* Fatigue*uparrow* Blood pressure↓ Irritability*uparrow* Aggressive behavior*uparrow*	(48, 53)
		Guanfacine-ER	Hyperactivity↓ Impulsiveness↓ Distractibility↓	Drowsiness*uparrow* Fatigue*uparrow* Appetite changes	(48)
	Neuropeptides	Oxytocin	Repetitive behaviors↓ Cognitive impairments↓ Core symptoms↓ Interpersonal communications*uparrow*	Delivery of small amounts of drug to the brain through nasal spray Nasal discomfort*uparrow* Skin problems*uparrow* Diarrhea*uparrow* Irritability*uparrow* Fatigue*uparrow*	(48, 54)
		Vasopressin	Interpersonal communications*uparrow* Social interactions*uparrow*	Ventricular arrhythmias, coronary ischemia, myocardial infarction, and high blood pressure in case of non-inhalation administration methods Bronchospasm, hyponatremia, angioedema, urticarial, peripheral vasoconstrictions, and local irritation at the injection site	(48, 55)
Non-pharmacological treatments	Complementary and integrative methods	Mental and physical activities	ASD-related destructive behaviors↓		(48, 56)
	Music therapy	Sung-word listening	Preserving the Frontal-temporal functional connectivity Mentalizing*uparrow* Regulate both emotions and moods Cognitive impairments↓ Modulating senses Modulating action-specific perception		(48)
	CBT		Anxiety↓ Depression↓ OCD↓ Disrupti ve behaviors↓ Core symptoms↓		(48)
	SBT	PECS	Improving emotional behaviors Social interactions*uparrow* Interpersonal communications*uparrow* Mental activity*uparrow* Independence*uparrow*		(48)
		Speech generating devices			
		Self-management			
		RIT			
	Dietary and nutritional supplements	Omega-3 fatty acids	Neurotransmission*uparrow* Oxidative stress↓ Inflammation↓ Immune system function*uparrow* Social interactions*uparrow* Attention deficits↓	Gastrointestinal problems*uparrow* Irritability*uparrow* Hyperactivity*uparrow* Stereotypic behaviors*uparrow* Social withdrawal*uparrow*	(48)
		Methyl B12	Improving redox balance Homocysteine levels*uparrow* Improving CGI-I score due to the increase in levels of methionine and reduction in S-adenosyl-lhomocysteine (SAH) Improving the ratio of SAM to SAH due to the improvement in cellular methylation capacity	No significant side effects have been reported	(57, 58)
		Vitamin D	Anti-inflammatory effects in the brain*uparrow* Regulating serotonin synthesis Improving the ABC, CARS, Autism Treatment Evaluation Checklist, and SRS scores		(57)
		Folinic acid	Improving neurological symptoms by regulating folate concentrations in cerebrospinal fluid Verbal communication*uparrow*	High levels of maternal plasma folate levels during pregnancy can be associated with high risk of ASD.	(57, 59)
		Camel milk	Anti-inflammatory effects*uparrow* Antibacterial effects on autistic enterocolitis *uparrow* Improving CARS score		(57)
		GFCF	Although in some studies, the use of GFCF has improved autistic behaviors, but in some trials, no significant difference was observed between the patient and control groups. As these two substances neither improve nor worsen the symptoms and this diet is an expensive diet, GFCF is not recommended as a main therapeutic approach for ASD	Social isolation*uparrow* Delay in the diagnosis of food allergy, celiac disease, or lactose intolerance*uparrow*	(57, 60)
		Probiotics	Gastrointestinal symptoms↓ Improving Total Behavior Problem score		(57)
		Digestive enzyme	Improving emotional response Improving general impression autistic score ASD-related behaviors↓ Gastrointestinal symptoms↓		(57)
		SFN	Improving ABC, SRS, and CGI-I scores	Insomnia*uparrow* Flatulence*uparrow* Constipation*uparrow* Weight gain*uparrow* Vomiting*uparrow* Diarrhea*uparrow* Aggression*uparrow* Seasonal allergies*uparrow*	(57, 61)
	Herbal medicine	*Panax ginseng*	Improving ASD-related abnormal behaviors		(62)
		Ukgansangajinpibanha granule	Aggressive behavior↓ Anxiety↓ Chronic pruritus↓ Insomnia↓		(63)

*ABC, Autism Behavior Checklist; ADHD, Attention deficit hyperactivity disorder; ASD, Autism spectrum disorder; CARS, Childhood Autism Rating Scale; CBT, Cognitive behavioral therapy; CGI-I, Clinical Global Impression Scale of Improvement; GFCF, Gluten-free and casein-free; Guanfacine-ER, Guanfacine extended release; PECS, Picture Exchange Communication System; RIT, Reciprocal imitation training; SBT, Social behavioral therapy; SFN, Sulforaphane; TER, Total effective rate.*

## Zebrafish as an Appropriate Animal Model for Autism Spectrum Disorder

Despite the advances in ASD pathophysiology understanding, there are still challenges in the accurate diagnosis and treatment of the disease (64). For instance, children who struggle with ASD represent heterogeneity in the phenotypic features (65). In addition, ASD is often accompanied by some disorders, such as attention-deficit/hyperactivity disorder (ADHD) (66), emotional and behavioral dysregulation (67), anxiety (68), and sleep disorders which can widen the spectrum even further (69). Moreover, the lack of adequate understanding and comprehensive knowledge of the disease (70), inadequate study design, and failure of clinical trial efficiency bring challenges to drug discovery and development pipelines (64). Hence, conducting preclinical animal testing can not only help decipher the complicated mechanisms underlying ASD but also pave the way for discovering newer and more efficient therapeutic approaches by developing a biological platform (71). Preclinical studies as a bridge between theoretical knowledge and clinical studies are of great importance in drug discovery and development pipelines. Since animal models are an integral part of this stage, observance of guidelines and ethical principles is one of the first and most important measures in preclinical studies. In this regard, the guidelines of preclinical studies establish some strict policies to ensure the observation of the highest standards and ethical principles of working with animals. For instance, a developing well-designed study and applying methods appropriate to the research goals, selecting animal models appropriate to the purpose of the study, employing a sufficient number of animals, taking any necessary measures to maintain animal welfare and safety, and trying to use alternatives to animal models, are among the most critical ethics that should be considered in animal studies (72). Regarding the mentioned ethical principles and international and regional guidelines, over the last decades, Zebrafish has attracted attention as a prominent animal model in various *in vivo* experiments (73), such as ASD studies due to its prominent neurological characteristics (74). Proper understanding of the zebrafish nervous system function as an outstanding animal model can be an important step in examining the various aspects of drugs and treatment approaches for disorders related to the human nervous system in drug discovery. It has been demonstrated that despite the obvious differences between Zebrafish and mammalians, relative neuroanatomical similarities in both CNS and peripheral nervous system (PNS) as well as some identical molecules in cell signaling pathways and genes involved in the function of the nervous system, have been observed in Zebrafish. Additionally, homologous structures of the brain such as cortex, amygdaloid complex, hippocampus, and basal ganglia have been created Zebrafish as a prominent *in vivo* system for discovering of drugs (75). In addition to some similarities between mammals and zebrafish, some difference has been reported. For instance, there is no midbrain dopaminergic populations (e.g., the substantia nigra and ventral tegmental area) in zebrafish (22, 76). However, zebrafish display behaviors attributed to these areas, which surprisingly are sensitive to dopamine (22, 77). When it comes to the optimal brain neurochemical properties of zebrafish, talking about associated neurotransmitters seems logical. In this case, it should be noticed that, the main neurotransmitter systems in mammals, such as GABA, glutamate, dopamine, norepinephrine, serotonin, histamine, and acetylcholine are presented in zebrafish (75, 78) allowing the researcher the opportunity to trust the result of animal model designing to an acceptable extent. This similarity has also been approved by pharmacological methods of neurotransmitter targeting (78–81). For instance, similar effects of drug usage on sleep between mammals and zebrafish were reported in a specific psychologic screening (78, 81). Neural regeneration researches also show that the mammalian CNS lacks any regenerative ability, so the number of treatment options available to overcome this limitation is few. In contrast, nervous system regeneration is a common feature among amphibians and also Zebrafish, which is a prominent animal model can repair neuropathies in PNS as well as damaged brain and spinal cord amputation in CNS. Analysis performed on Zebrafish shows that both CNS and PNS have a high capacity for neurogenesis, regeneration of the spinal cord, and re-growth of the axon in the damaged area where glial cells play an important role in the axon targeted orientation and correct synapse formation with postsynaptic cells (82, 83). If a scientific view is taken from the anatomical level of the zebrafish brain and nervous system to the psychological level of the fish, it can be concluded that due to some genetic and anatomical similarities, this animal model can be expected to perform some social behaviors in interaction with the surrounded environment or other living organisms. It has been demonstrated that as social behaviors are an important part of human brain function defects in these behaviors can be considered symptoms of ASD. In this regard, zebrafish sociality studies indicated that the larvae, juvenile, and adult zebrafish have been able to open new doors to the study of social behaviors associated with ASD, such as social preference, social plasticity, social inference, social recognition, shoaling, mimicry, aggression, and restricted or repetitive behaviors (84). To promote the research in this field, some of the most common tests to examine the social behavior of zebrafish are discussed as detailed below.

### Social Preference Test

The Zebrafish social preference test refers to tests that examine social attraction and behavioral disturbances of animal in relation to social stimuli with aim of promoting translational medicine and drug discovery and development pipelines in neurological diseases researches. In Zebrafish modeling condition, the social preference tests are performed in two stages, including habituation (fish swimming in the test environment alone) and communication with a social stimulus (placing one or two real populations of the same species, using recorded videos of fish movement, or using virtual reality in the adjacent chambers). The amount of time spent near stimuli is a measure of fish social preference, which is obtained by recorded videos of fish movement in the test environment. It should also be noted that, the consideration of effects of physiological traits and interpersonal behaviors in both test and stimuli fish is necessary (85). For instance, in 2020, Landin et al. indicated that the zebrafish oxytocin system can regulate social behavior, which was indicated *via* treatment of L-368,899 as an inhibitor of Zebrafish oxytocin receptors (86). In addition, attention to environmental factors are obviously of great importance for studying social preference in Zebrafish (85).

### Shoaling Test

Shoaling refers to the behavior that a fish performs by being in a population of its kind to receive some benefits such as protection from the predator, forage finding, and successful mating. To study the shoaling behavior, the target Zebrafish is placed in a tank with a group of its kind. Hence, the measurement of distances between the target and other Zebrafish can indicate different aspects of shoaling (87). In recent years, various studies have been conducted to investigate how shoal behavior is modeled in Zebrafish. For instance, in 2010, a study by B et al. showed that applying 1% ethanol concentration could prevent shoal behavior in Zebrafish models, whereas low concentrations of ethanol (<1%) stimulate the emergence of shoaling (88). In another study conducted in 2012, the effect of hallucinogenic drugs, including phencyclidine (PCP) and mescaline was studied on Zebrafish shoal behavior. The results of this study indicate that shoaling was tighter at 20 mg/l mescaline in comparison to the control group. However, PCP did not affect the emergence of Zebrafish shoal behavior in this experiment (89). In addition to the above, a recent study performed two-dimensional and three-dimensional analyzes of Zebrafish’s behavior after treatment with two anxiogenic drugs, including conspecific alarm substance (CAS) and caffeine (CAF), and an anxiolytic drug including diazepam (DZP). The specific results of this experiment indicate that both CAS and CAF have reduced the volume of shoaling. Additionally, as a result of treatment with these two drugs, the rate of shoal geotaxis has increased and zebrafish got closer to the centroid point. Moreover, the effect of CAS, not CAF, formed a tight shoal. However, in contrast to CAS and CAF, DZP had positive effects on shoal behavior (90). Regarding shoal behavior investigation in Zebrafish, it is noteworthy to mention that some factors such as sex, phenotype (91), age (92), group size, test area space (93), and water flow should also be considered (94).

### Inhibitory Avoidance Test

This type of test is generally used to analyze learning and memory processes based on training and/or testing of animals to evaluate the relationship between brain and behavior. The test consists in placing the Zebrafish inside the black and white box and tracking the Zebrafish’s reaction to receiving an electric shock from the device embedded in the black section for many days. Any delay in the entry of fish from the white section to the dark part in the following days after receiving the shock indicates that the avoidance behavior has formed in the model. The results obtained from the study by Manuel et al. in 2014 showed that both electric shock and the emergence of inhibitory avoidance lead to increase in cortisol levels of the Zebrafish to cause flight and fight response. However, upregulation of genes associated with mineralocorticoid receptor (nr3c2), glucocorticoid receptor alpha [nr3c1 (alpha)], and cocaine- and amphetamine-regulated transcript (cart4) is observed only in the case of receiving electric shock (95). In recent years, various studies have been performed to investigate the effects of different compounds or conditions on the quality of Zebrafish inhibitory avoidance. For instance, Amorim et al. conducted investigations in the field of the effects of alcohol on the inhibitory avoidance paradigm in the Zebrafish model. Although the result of this experiment showed the ineffectiveness of acute alcohol exposure in inhibitory avoidance behavior, the authors of this study stated that chronic consumption of alcohol and then quitting can lead to learning impairment (96). But in another study, Manuel et al. focused their study on the separate effect of age, Zebrafish habitat enrichment, and the effect of both of these factors on inhibitory avoidance behavior. In this experiment, cellular and molecular studies have shown that 6- and 12-month-old fish have less inhibitory avoidance behavior due to reduced mRNA expression in some telencephalon-related genes, including *pcna*, *neurod*, *cart4*, and *cnr1*, and increased in *nr3c2/nr3c1*(α) *and nr3c1*(β)*/nr3c1*(α) ratio. However, such a decrease in this behavior has not been observed in 24-month-old fish. Instead, 24-month-old fish showed delays in avoidance behavior due to decreased expression of three genes, including *bdnf*, *pcna*, and *cart4* (97). In the same year, another study showed that giving food as a reward to fish could change the behavior of inhibitory avoidance. In this experiment, like the previous study, a decrease in the expression of the *cnr1* gene associated with Zebrafish telencephalon has been discovered. However, this study reported the overexpression of *crf* gene in this model. Based on these cellular and molecular findings, the authors stated that Zebrafish feeding, as a reward, could reduce the behavior of inhibitory avoidance after the first electric shock (98).

### Aggression Test

Aggression can be categorized as an adaptive and complex behavior in Zebrafish model. Aggressive behavior can be observed in the Zebrafish with signs such as raising the fin, approaching, biting, wave-like body motion, opening the mouth, changing in body pigmentation, swimming in a circular direction, charging, and chasing to defend and show courage and bravery against the enemy (99). To investigate this type of behavior, various techniques, such as an inclined mirror, two flat mirrors with different acclimation periods, live conspecific stimulus, clay-model stimulus, and video stimulus are used in Zebrafish. In 2015, Way et al. examined the effects of using the six mentioned techniques and stated that two behaviors, including bite and darts, were more pronounced in the flat mirror or live conspecific and inclined mirror stimulus, respectively (100). It should also be noted that consideration of the effect of some environmental factors, such as the enrichment of laboratory tank is of great importance for aggressive behavior studies in Zebrafish models (101).

### Repetitive Behavior Test

**FIGURE 2:**
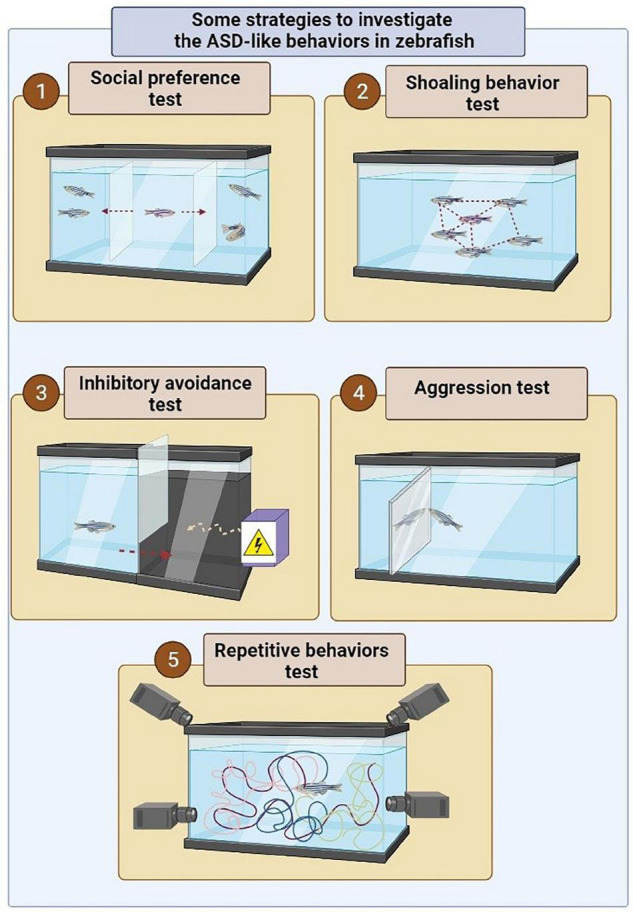
Some strategies to investigate the ASD-like behaviors in zebrafish. (1) In order to assess social preferences, a live zebrafish or robot or photo or zebrafish video is used in an adjacent tank or multi-chamber tank to show the conspecific fish. In this experiment, the social preference behavior is examined by measuring the distance of the fish from the conspecific fish (photo, video, or robot) in the adjacent tank. (2) In order to investigate shoal behavior in the zebrafish, the studied model is placed among a group of conspecific fish inside a tank. In this experiment, the incidence of shoal behavior is determined based on the average distance between the fish in a tank. (3) In order to investigate behavioral avoidance, one of the chamber of the tank becomes dark, which is instinctively more desirable for zebrafish than a brighter environment. However, the presence of the electric shock in a dark environment is considered a risk factor for zebrafish. Hence the conflict between swimming into the dark environment instinctively and avoiding electric shock can be used to investigate behavioral avoidance. (4) In order to assess the aggression behavior, a mirror is applied inside the tank of the zebrafish. (5) The locomotor behavior can be assessed by tracking the position of zebrafish in 3D space and swim path characteristics (22).

Repetitive behaviors known as a symptom of neuropsychiatric disorders such as ASD that can also be modeled in animal models such as Zebrafish. Generally, the examining repetitive behavior in Zebrafish is based on the use of novel tank test and tracking the movement of Zebrafish in the studied environment. However, inducing the repetitive behavior can be done through various modeling approaches in Zebrafish populations. For instance, in 2018, Liu et al. revealed that CRISPR/Cas9-induced shank3b mutant zebrafish show abnormal repetitive movements in Zebrafish (102). In addition to the use of genetic techniques, the use of some compounds, such as quinpirole (103), ketamine (104), and ibogaine can induce the repetitive behavior in Zebrafish model (105). However, a study indicated that exposure to moderate levels of ethanol can reduce such behaviors in Zebrafish (Figure 2) (106).

## Autism Spectrum Disorder Modeling Methods in Zebrafish

### Genetic Manipulation Techniques

Zebrafish is considered a suitable animal model for studying genetic diseases due to the high orthology of the genome with humans. According to the results obtained from the Zebrafish genome sequencing project in 2001, zebrafish share about 70% of human genes. In contrast, 69% of human genes have the orthology to those of zebrafish (107). In addition, 82% of human disease-associated genes have orthologues in zebrafish. It should also be noted that more genetic data are also online available in the ZFIN database^1^ (ZFIN is a comprehensive authoritative resource of zebrafish genetic and genomic data which can be a considerable step toward enhancing the insight into human disease processes) (108). Regarding ASD pathophysiology, genetic studies imply that zebrafish share about 62% of the 858 genes involved in human ASD, which is significantly valuable for preclinical studies. To promote research on the ASD and also pave the way for discovering new therapeutic approaches, the Simons Foundation Autism Research Initiative, or SFARI for short, created the gene loss-of-function lines with mutations in 12 ASD risk genes of zebrafish, which have been described as detailed in the Table 2 (109).

**TABLE 2:** Mutations in 12 ASD risk genes of zebrafish created by SFARI.

Gene name	Natural function of gene	Some results of abnormal function of mutated gene in zebrafish ASD model	References
ARID1B	Controlling and regulation of neurite outgrowth Suppressing Wnt/β-catenin signaling pathway	The imbalance of osteogenic and chondrogenic gene expression patterns Disturbance in the Wnt/β-catenin signaling pathway	(110, 111)
CHD8	Reduction in expression of genes related to Wnt/β-catenin signaling pathway	Macrocephaly Impairment in gastrointestinal motility	(112–114)
FMR1	Regulation of synaptic plasticity Regulation of neuronal protein production	Hyperactivity Anxiety like behavior Impaired inhibitory avoidance Alteration in Synaptic plasticity by reduction in long-term potentiation Craniofacial abnormalities	(115–117)
MECP2	Regulation of transcriptional repression or activation Regulation of RNA-splicing Regulation of brain function Development of neurons	Defective thigmotaxis Augmented tactile evoked potentials Low motor activity Excitation/Inhibition unbalance Decreased activity and anxiety-like behavior Shorter lifespan	(118, 119)
PTEN	Suppressing tumor Regulating metabolic pathways Regulating of neuron size	Hyperplasia Dysplasia High angiogenesis High *vegfaa* expression Abnormal head, tail, and notochord shapes Impaired formation of vascular network and inner ear development High levels of phosphorylated Akt Low yolk extensions	(120–123)
CNTNAP2	Cell adhesion molecules and receptors in nervous system Synaptic regulation Regulating the neuronal synchrony	Hyperactivity at nights Hypersensitivity to drug-induced seizures	(124, 125)
DYRK1A	Regulation of the growth and development of nervous tissue Increase in dendritic arborization and axon branching Neuronal trafficking Aging	Reduction in the strength of relationships and the sense of solidarity among members of a community Increasing anxiety Microcephaly Reduction in stress responses produced by hypothalamus	(126)
GRIN2B	Neural formation	Needs further investigation	(109)
NRXN1	Synapse organization Modulator of the strength of neuroligin–neurexin interactions Regulation of calcium channel activity	Reduction in blood flow Angiogenesis Low thigmotaxis Developing vain tail size Deformity of caudal vein Vascular malformation Disturbance of locomotor activity	(127, 128)
SCN2A	Encoding the voltage-gated sodium channel Regulation the action potential initiation and downstream axonal propagation	Hyperactivity Spontaneous activity of the electrograph Seizure-like behaviors Abnormal neuronal firing	(129, 130)
SHANK3	Functioning and formation of synapses Encoding scaffolding proteins at postsynaptic densities	Reduction in gastrointestinal motility Reduction in peristalsis Reduction in serotonin-expressing enteroendocrine cells Abnormal morphology Displaying repetitive behaviors Difficulties in social interactions Reduction in the number of differentiated neurons	(102, 131, 132)
SYNGAP1	Regulating of synaptic plasticity Development and proper synapse function Dendritic spine development and maturation Encoding a brain-specific synaptic Ras GTP-ase activating protein	Reduction in cell survival in the midbrain, hindbrain, and spinal cord Abnormal morphology of the brain Displaying seizure like behaviors Reduction in the number of GABAergic neurons in the midbrain and hindbrain Reduction in the number of excitatory neurons in the hindbrain	(133, 134)

*CRISPR/Cas9, clustered regularly interspaced short palindromic repeats and CRISPR-associated protein 9; ENU, N-ethyl-N-nitrosourea; TALEN, Transcription activator-like effector nucleases; TNF-α, Tumor Necrosis Factor-α; ZFN, Zinc finger nucleases.*

The increase of interest in the Zebrafish model was in parallel with the development of various disease modeling methods. In this regard, to achieve a comprehensive knowledge of the mechanisms involved in Autism spectrum disorder and evaluate the effects of different candidate drugs, various genetic methods, including N-ethyl N-nitrosourea (ENU), transcription activator-like effector nucleases (TALEN), clustered regularly interspaced short palindromic repeats and CRISPR-associated protein 9 (CRISPR-Cas9), Targeted Induced Local Lesions in Genomes (TILLING) method, and zinc finger nucleases (ZFN), have been applied in zebrafish researches, which are discussed as detailed below (78):

#### N-Ethyl N-Nitrosourea

N-ethyl N-nitrosourea is a mutagenic chemical used in both forward and reverse genetics approaches. The function of ENU is based on generating random point mutations in the genome of the target organism (135). Over the past years, ENU-generated Zebrafish have been employed as one of the animal models in the search for ASD mechanisms. For instance, in 2009, Marjo et al. focused their study on Fragile X syndrome (FXS), which is caused by a mutation in a gene called *FMR1*. As a result of mutations, in gene *FMR1*, intellectual and developmental disabilities and some behaviors, such as hyperactivity, seizures, and autistic behaviors develop in the individuals. In this experiment, two *FMR1* knockout alleles (hu2787, hu2898) in zebrafish were generated by combining ENU and TILLING methods. As a result of this experiment it was revealed that mRNA derived from the knockout hu2787 allele in zebrafish is not as stable as the normal type. Also, the expression of Fmr protein has increased in some regions of the brain, including the cerebellum and the telencephalon. In addition, they showed that mutant zebrafish are capable to live and showed no signs of craniofacial abnormalities or defects in neurite branching in Rohon–Beard neurons (136). Similar to the previous study, In addition, another study has been performed on the integration of ENU with TALENs to investigate the autism-associated gene *neurobeachin (nbea*) function and the relationship between the formation of electrical and chemical synapses (137). Moreover, in other studies the integration of two ENU and CRISPR methods have also been conducted to investigate stereotyped behaviors, anxiety, hyperactivity (138), shoaling (139), atypical behaviors, learning and memory disorders, and craniofacial abnormalities in zebrafish (140). Furthermore, the other study focused on the assessment of Rett syndrome, as a neurological disorder with autism-like features, *via* applying the combination of two ENU and the morpholino methods (141). Although the method of using ENU is recognized as one of the common methods in the study of animal models such as zebrafish, this method is neither low cost nor effortless. Therefore, a method called TILLING was introduced to identify mutations with fewer issues (142).

#### Targeted Induced Local Lesions in Genomes

Targeted Induced Local Lesions in Genomes method is considered one of the reverse genetic strategies that detect specific mutations in target genes by combining chemical mutagenesis (like ENU) with a high throughput screening (HTS) method. In order to use the TILLING approach in zebrafish studies, a large library of ENU-mutagenized zebrafish populations is needed, which can be prepared from one of two strategies, including (1) cel1 enzyme and (2) resequencing methods (143). Over the past years, the use of TILLING method has also helped to model ASD and related symptoms in zebrafish. For instance, in 2013, Pietri et al. applied the TILLING methods to advance their research in ENU-mutagenized zebrafish for ASD by investigating abnormalities of evoked somatosensory and abnormal thigmotaxis through the *mecp2* gene analyzing (119). Despite the advantages of TILLING, there are several disadvantages. For instance, in comparison to other genetic modeling strategies, the performing TILLING method requires considerable effort and time in zebrafish-based preclinical studies. Because many human genes may have two zebrafish orthologs due to the duplicated genome of zebrafish. In addition, the probability of finding the desired mutations by this method is low (78). Therefore, to overcome these problems, some methods such as transcription activator-like effector nucleases (TALEN) and zinc finger nucleases (ZFN) have been introduced as two novel gene-editing methods, which can have a major contribution to making rapid gene modification possible (78, 144–147).

#### Zinc Finger Nucleases

Zinc finger nucleases is recognized as DNA binding proteins with the capability of generating double-strand breaks in target DNA by their DNA-cutting endonuclease domain fused to zinc finger domains (148). Over the past years, the ZFN approach could also provide the opportunity to promote the knowledge of different disorders such as ASD as a potential strategy for disease modeling and drug discovery. In 2017, an investigation of the role of the *CNTNAP2* gene with ASD was presented by Hoffman et al. to investigate phenotypic suppressors and pathways with the intention to discover new therapeutic approaches through generating mutant zebrafish. The authors indicated that the loss of function in the *CNTNAP2* gene can lead to dysfunctionalities in GABAergic neurons as well as GABAergic and glutamatergic signaling pathways. In this study, a high rate of sensitivity to drug-induced seizures and hyperactivity during the nighttime was also reported. Accordingly, it can be stated that the *CNTNAP2* gene can be used as an effective drug target for the treatment of ASD (124). It is worth mentioning that like any other method, the ZFN technique has some cons as well as pros. In this regard, evidence indicated that ZFN engineering requires high expertise. In addition, there are fewer target sites for the ZFN approach. Moreover, the ZFN approach is accompanied by more off-target mutations which can pose difficulties to preclinical studies (148).

#### Transcription Activator-Like Effector Nucleases

Transcription activator-like effector nucleases method refers to one of the genome editing techniques that is used to create cuts and mutations in specific sequences of DNA of the organism genome by performing endonuclease activity. According to studies on the effectiveness of the TALENs technique in zebrafish, the mutation rate in both somatic and germ cells of zebrafish have been reported to be dramatically high. Accordingly, it can be considered that TALENs method is one of the effective methods in the Zebrafish modeling process (149). Similar to the previous methods mentioned, the TALENs method has also been used as an effective approach to assessing the symptoms associated with ASD. For example, in 2017, the major role of *DYRK1A* mutation as a factor involved in the emergence of autistic or intellectual disability induced by microcephaly in Down syndrome was investigated by Kim et al., in which the target gene was mutated through the TALENs method in zebrafish (150). In addition, another study conducted in 2020, examined the link between the oxytocin signaling and social behavior associated with ASD including, social preference and social recognition through generating zebrafish mutants with mutations in the *oxtr* gene by the TALENs method (151). The comparison between TALENs with ZFN implies that the design of TALENs for various researches is more facile than ZFN. TALENs design is also possible in a shorter time with different lengths of TALE repeat arrays. In addition, the selection range of the target site in TALENs method is wider than ZFN. Moreover, the rate of off-target mutations is lower than ZFN. Furthermore, the results of one study showed that depending on the type of ZFN consumed, ZFN-induced cytotoxicity is more likely. However, it is worth noting that the TALENs technique, also has some limitations and disadvantages in addition to its advantages and high efficiency in zebrafish modeling. For instance, the cDNA encoding the TALENs is much larger than that of ZFN, which can complicate therapeutic research based on the TALENs. In addition, the TALENs delivery by viral vectors remains a challenge due to the presence of the repeat array in TALENs (148).

#### CRISPR/Cas System

Compared to other genetic modeling methods, it can be declared that CRISPR/Cas technique is one of the newest and most advanced genome editing technologies in the bench to bedside studies (148, 152). Functionally, CRISPR/Cas system is based on generating double-strand breaks into the target DNA, by the cooperation of two complementary sequences of CRISPR and Cas and their endonuclease activities (148). Over the past years, the high efficacy and versatility of the CRISPR technique have gained interest in applying this genome editing approach in research based on zebrafish modeling (153), particularly in neurological disorders such as ASD. For instance, in 2018, Liu et al. published a paper in which they developed zebrafish with mutations in the *SHANK3* gene to indicate the association of the mutated *SHANK3* with autism-like behaviors (102). In addition, in 2020, Ruzzo et al. conducted investigations in the field of the rare genetic variants’ impacts on ASD development by applying whole-genome sequencing to a wide spectrum of families with autistic children. They also revealed that the mutation in the *nr3c2* gene, which has been conducted through CRISPR/Cas9 in zebrafish, can lead to disruption in social interactions and sleep difficulties (154). Regrading to CRISPR-Cas9, it should be noted that CRISPR can perform the gene modification process in a more efficient way than the two previous ones. In a simple expression, a single guide RNA (sgRNA) with the mRNA encoding Cas9 enzyme is directed into the zebrafish embryo, and the Cas9 enzyme can make DNA cleavage in a way that makes insertion-deletion mutations. Therefore, in comparison to previous methods, this technique is easy to use as well as its widespread accessibility to many laboratories (78).

#### Morpholino

In addition to the above, morpholinos is another common genetic method in zebrafish modeling. Nowadays, because of low cost, ease of use, and a few numbers of rapidly generating genetic mutants, a large number of studies are using morpholinos to repress a specific gene functioning. In detail, morpholinos is a reverse genetic method (21, 78, 155), in which the mRNA splicing or translation processes can be blocked with the intention to knock down a target gene (78, 156, 157). If the applications of morpholino expanded to ASD disease, many related studies can be found in this regard such as studies on the *ARX* gene for assessment of intellectual disability (158), *AUTS2* gene (159), *kcnj10a* gene (160), *cep41* gene (161), *ctnnd2b* gene (162), *syngap1b* gene, and *shank3a* gene for evaluating ASD–associated behaviors (134).

However, there are some shortcomings in the use of morpholinos such as inducing transient effects and the ability to generate off-target effects (78, 163, 164). Another drawback of the morpholino modeling method is the activation of p53, which can trigger the apoptosis pathway and subsequently, create adverse effects such as changes in head size or brain structure (78, 165, 166). In order to efficiently use the morpholino technique, two control items have been explained: (1) the necessity of using two different morpholino targeting sites, and (2) the warranty to rescue the phenotype by using mRNA lacking the morpholino target site (78, 165). Based on some studies, using these controls doesn’t give an equivalent result to gene mutation (78, 167). In order to provide this equality, a new guideline has been introduced, which makes requires morpholino-induced phenotypes to be confirmed in genetic mutants. Nowadays, CRISPR makes this necessity reachable (78, 167). It is worth mentioning in mind that concerning morpholinos application in preclinical studies, it is important to follow the guidelines in the future due to the probable non-specific effects of morpholinos on zebrafish models which can make the phenotype interpretation challenging (78).

### Non-genetic Manipulation Techniques

In addition to genetic methods, the use of non-genetic methods is considered one of the effective approaches in zebrafish modeling to scrutinize the mechanisms involved in ASD development and discover efficient drugs through drug discovery and development platforms. According to the pathophysiology of ASD, current non-genetic methods in modeling ASD can be divided into two groups chemical and non-chemical approaches (109). One of the known chemical methods in creating zebrafish models for ASD is valproic acid (VPA). In a recent study conducted by Joseph et al., the effect of VAP on the development of ASD-like behaviors was investigated in zebrafish. As a result of this study, it was found that zebrafish treated with VPA have low viability. It was also reported that the dechorionation of larvae has decreased. In addition, some morphological and anatomical alterations were observed in the zebrafish. For instance, examinations indicate that the tail and the spinal cord are deformed. Moreover, the lack of swim bladder and the incidence of pericardial edema have been observed in zebrafish. However, the most important results were related to the occurrence of some ASD symptoms including hyperactivity and social communication problems. Regarding the results obtained from the study, Joseph et al. applied Duloxetine (DLX) as a candidate drug for alleviating the ASD symptoms which led to significant results. For instance, DLX could improve hyperactivity, social interactions, and anxiety. In addition, at the cell and molecular level, DLX was succeed in the regulation of the acetylcholinesterase -activity and the PI3K/AKT/mTOR pathway (168). In addition to VAP, Pentylenetetrazole (PTZ) is another chemical compound that can be effective in modeling ASD-like behaviors in zebrafish. In order to investigate the effects of PTZ on zebrafish and to discover new therapeutic approaches, in 2015, Torres-Hernández et al., conducted a study. In this regard, zebrafish were first immersed in a tank containing valerenic acid, valerian extracts, or antiepileptic drugs (AEDs). After treating the fish with the mentioned drugs, the zebrafish were placed in a tank containing PTZ. As a result of this experiment, it was determined that the zebrafish displayed stereotyped behaviors and PTZ-induced seizures after PTZ treatment. However, valerenic acid and valerian extracts could postpone the onset of PTZ-induced seizures. In addition, both valerenic acid and valerian extracts had an effective interaction with clonazepam in this process. It should also be noted that the action to delay the onset of PTZ-induced seizures by Valerian extracts has been increasingly better than the function of valerenic acid (169). Regarding non-chemical approaches, the novel tank test is one of the common modeling approaches, which have been developed and introduced over the past years to model anxiety-like behavior in zebrafish (170–173). According to a study conducted in 2019 by Haghani et al., the zebrafish examined by the novel tank test demonstrated the anxiety-like behaviors and a reduction in erratic swimming or darting. In addition, a different rate of acclimation and high levels of thigmotaxis in the light conditions were observed between different ages of zebrafish models (174). Since this non-chemical strategy has been successful in causing anxiety-like behaviors, some researchers have investigated the effects of drugs on zebrafish modeled by the novel tank test. For instance, Levin et al. introduced the Nicotine as a therapeutic approach for relieving the anxiousness induced by the novel tank test in zebrafish. As a result of this study, it was found that nicotine-treated zebrafish exhibit behaviors such as decreased diving to the bottom of the tank. In addition, an increase in locomotor activity, exploration, and two-point discrimination learning were reported, which indicated a reduction in the anxiousness of zebrafish models (175). In recent years, the association between the gastrointestinal tract and the brain has been cited as one of the key mechanisms involved in the pathogenesis of ASD. In this regard, the prominent similar characteristics of zebrafish gastrointestinal tract with those of mammalians, such as having some similar main cells in the gastrointestinal tract, highly conserved gastrointestinal tract function and physiology, innervation by the enteric nervous system, and expression of some similar genes involved in the structure and function of the gastrointestinal system have made the Zebrafish as a suitable animal model for evaluating studies on diseases or disorders related to the gastrointestinal system or gut-brain axis (176). Since gastrointestinal disorders can be categorized as one of the symptoms of ASD, in 2019 James et al. conducted a study based on the effect of a SHANK3 mutated gene on gastrointestinal distress. In this study, they initially developed the Phelan-McDermid syndrome (PMS) model of zebrafish *via* producing *shank3ab*Δ*C*± *and shank3ab*Δ*C−/−* mutants by applying the CRISPR/Cas9 technique. As a result of this experiment, a reduction in peristaltic contractions and an increase in passage time of compounds in the gastrointestinal tract of *shank3ab*Δ*C* ± mutant Zebrafish were reported, while the number of serotonin-positive enteroendocrine cells (EECs) in both mutant models decreased. It should also be noted that the larvae which were developed after injection of the transcript of human SHANK3 isoform into *shank3ab*Δ*C*± mutant Zebrafish, could empty their intestinal bulb-like wild type. However, the posterior motility of their intestine was still impaired (132). In addition to the above, many studies have also focused on the investigation of the gut-microbiota-brain axis in the development of ASD (177–179). To promote the research in this regard, the use of the germ-free (GF) approach and the application of Gnotobiology science have been expanded in the Zebrafish model. Due to the high growth rate and transparent body of zebrafish, the researchers can easily process them and established them as GF zebrafish models for ASD. Although the use of GF zebrafish models in studies still needs further investigation, some observations have been found that indicate a link between microbiota and the occurrence of social behaviors in the organism (109).

## Conclusion

Autism spectrum disorder is recognized as one of the childhood neurodevelopmental disorders, which are accompanied by difficulties in social communications and interactions as well as displaying atypical behaviors and intellectual disability. Due to the symptoms and disorders associated with ASD, major challenges are faced by patients. On the other hand, there is insufficient understanding of the mechanisms involved in the pathophysiology of ASD due to the complexity of the disease. Accordingly, detailed studies of the mechanisms underlying ASD can pave the way for discovering new approaches with high therapeutic potential to maximize the quality of life of patients with ASD as well as their caregivers (180). In recent years, to promote the studies, zebrafish has been used as a leading model in neurological disorders and injuries research (73, 181) such as ASD to investigate the pathophysiology of disease and to evaluate the potency of candidate drugs (22). However, the use of animal models in preclinical studies is still controversial. Because the use of animals in scientific studies requires the observance of strict ethical principles (72, 182–184). Thus, in recent years, studies in the field of neurological disorders have led to the use of new technologies such as organ-on-a-chip. Since this strategy is based on the use of the patient’s stem cells, some of the issues accompanied by applying animal models during the drug discovery and development platforms can be almost addressed and the studies can be upgraded to a more accurate level (183). Moreover, the application of new technologies such as artificial intelligence (AI) and its subsets (e.g., machine learning) as cutting-edge technologies could revolutionize biomedical studies, particularly ASD diagnosis and treatment (185–187). Even, some studies have even directly examined the behaviors of people with autism using advanced technologies. For example, in a recent study, the use of software to record children’s dragging patterns was suggested. In this experiment, the coordinates of the dragging patterns of 60 children were recorded by working with a tablet and related software. This study showed that the analysis of recorded patterns using supervised machine learning algorithms has been able to diagnose autism with 93% accuracy in children (188). In other studies, the application of autism diagnosis approaches using computer-aided eye tracking (189) and automatic facial expression recognition software is suggested, which can facilitate more accurate and better examination of emotions and thoughts expressions. Therefore, it can be concluded that the introduction of computing technologies and computer software can be revolutionized the timely diagnosis of neurological diseases such as ASD in the near future (190).

## Author Contributions

AT-B, SH, MA, and HS drafted the manuscript. MT, NN, and BL participated in the study design and interpretation and finalized the manuscript. AT-B and BA supervised the project from a scientific view of point and advised on the study design. All authors read, provided feedback, and approved the final manuscript.

## Conflict of Interest

The authors declare that the research was conducted in the absence of any commercial or financial relationships that could be construed as a potential conflict of interest.

## Publisher’s Note

All claims expressed in this article are solely those of the authors and do not necessarily represent those of their affiliated organizations, or those of the publisher, the editors and the reviewers. Any product that may be evaluated in this article, or claim that may be made by its manufacturer, is not guaranteed or endorsed by the publisher.

## Footnotes

1
https://zfin.org/
